# Single-Level Decompression for Extensive Spinal Epidural Abscess: A Case Report and Literature Review

**DOI:** 10.7759/cureus.108745

**Published:** 2026-05-12

**Authors:** Zenab Sher, Rudra N Shah, Maria Imtiaz, Hafiz Raza, Ashraf Elmahadi, Rahim Hussain

**Affiliations:** 1 Neurosurgery, University Hospitals Coventry and Warwickshire NHS Trust, Coventry, GBR; 2 Plastic Surgery, Queen Elizabeth Hospital Birmingham, Birmingham, GBR; 3 General Practice, Hafiz Healthcare Ltd, Birmingham, GBR

**Keywords:** catheter-directed irrigation, extensive spinal epidural abscess, holospinal epidural abscess, skip laminectomy, spinal epidural abscess

## Abstract

Extensive spinal epidural abscess (SEA), characterized by multilevel or holospinal involvement, is an uncommon but potentially devastating spinal infection associated with significant neurological morbidity. Due to its rarity, optimal surgical management remains debated, with most evidence derived from case reports and small case series. We present an illustrative case of extensive SEA managed surgically and contextualize the management strategy through a review of the available literature. A 58-year-old male patient with an extensive SEA involving multiple spinal levels from cervical to lumbar presented with clinical and radiological features consistent with infection and neurological compromise. MRI demonstrated a multilevel epidural purulent collection from C2 to L3, requiring urgent surgical intervention. The patient underwent limited decompression via a single-level laminectomy combined with catheter-directed epidural irrigation to achieve adequate drainage while avoiding extensive multilevel laminectomies. *Staphylococcus aureus* was isolated from intraoperative samples, and targeted antimicrobial therapy was administered postoperatively for six weeks. The patient demonstrated neurological improvement following surgery and achieved complete recovery at follow-up. A systematic search of MEDLINE, EMBASE, Scopus, the Cochrane Library, and Google Scholar was conducted in accordance with the PRISMA 2020 guidelines. Studies published between 1991 and 2025 describing surgically treated extensive or holospinal SEA were included. Thirty-four studies met the inclusion criteria. Patients were predominantly male (approximately 70%), with diabetes mellitus being the most common comorbidity, and *S. aureus* the most frequently isolated pathogen (reported in approximately 60% of cases with microbiological data). Most reported cases utilized limited or skip decompression with catheter-directed epidural irrigation to minimize the need for extensive multilevel laminectomy, with neurological improvement reported in the majority of patients (greater than 80% across included studies). Extensive SEA can be effectively managed using targeted decompression combined with catheter-directed irrigation and prolonged antimicrobial therapy. This case highlights the feasibility of limited decompression strategies in achieving adequate abscess drainage while reducing surgical morbidity, though conclusions should be interpreted in the context of retrospective, heterogeneous case-level data.

## Introduction

Spinal epidural abscess (SEA) represents a neurosurgical emergency with the potential for rapid neurological deterioration and irreversible disability if diagnosis or treatment is delayed [1,2]. The overall incidence of SEA has risen over recent decades, with reported rates increasing from approximately 0.2-2.8 cases per 10,000 hospital admissions, likely reflecting an aging population, a rising prevalence of immunosuppression, and improved access to advanced imaging [3]. Extensive or holospinal SEA remains rare, accounting for a small subset of all SEA cases. This entity is characterized by continuous or near-continuous epidural infection spanning multiple spinal regions and is associated with a higher burden of neurological compromise at presentation [4].

Early clinical features are often nonspecific, with back pain and systemic symptoms frequently preceding neurological deficits. The classic triad of fever, spinal pain, and neurological impairment is observed in fewer than 40% of cases, contributing to diagnostic delay [1]. Surgical management should be considered when conservative criteria are not met, specifically in the presence of neurological deficits, progressive symptoms, failure of medical therapy, or abscess extent involving three or more spinal regions. MRI with gadolinium enhancement is the diagnostic modality of choice, allowing accurate delineation of abscess extent, spinal cord compression, and associated osteodiscitis or paraspinal infection [3,5].

Management strategies for SEA depend on neurological status, medical comorbidities, and abscess extent. While select patients without neurological deficits may be managed conservatively, surgical intervention is generally recommended in the presence of neurological impairment, extensive disease, or failure of medical therapy [6]. Traditional multilevel decompressive laminectomy for extensive SEA is often impractical due to prolonged operative time, increased blood loss, postoperative spinal instability, and greater perioperative morbidity, with reported complication rates exceeding 30% in some series [7].

A systematic review and literature search (conducted in accordance with the PRISMA and the Peer Review of Electronic Search Strategies (PRESS) guidelines [8,9]) aims to synthesize available evidence on surgically managed extensive SEA, with particular emphasis on operative strategies, irrigation techniques, and neurological outcomes, and to illustrate contemporary management through a representative clinical case.

Consequently, alternative surgical strategies employing limited or skip decompression combined with catheter-directed epidural irrigation have been increasingly described [10-14]. Despite growing adoption of these approaches, there remains no consensus regarding optimal operative management of extensive SEA.

## Case presentation

A 58-year-old man presented with a several-day history of fever, progressive lower-limb paresthesia, and urinary retention. His medical history was notable for prolonged corticosteroid therapy, placing him at increased risk of infection. On presentation, he reported worsening lower back discomfort accompanied by bilateral lower-limb sensory disturbances and difficulty voiding.

Neurological examination demonstrated bilateral lower-limb weakness with associated sensory disturbances, while upper-limb function remained intact. The patient was febrile, and laboratory investigations revealed markedly elevated inflammatory markers (C-reactive protein 187 mg/L, white blood cell count 14.2 × 10⁹/L) consistent with an underlying infectious process.

**Figure 1:**
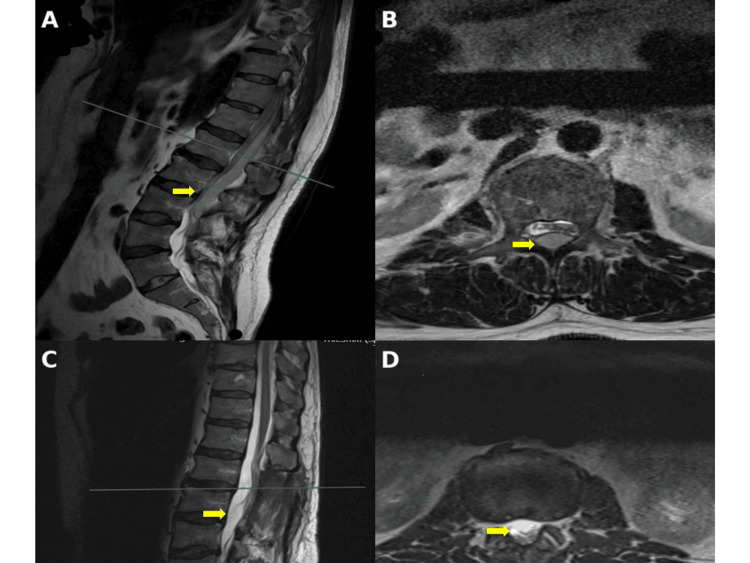
Preoperative and postoperative MRI findings in a 58-year-old male patient with extensive SEA (A) Preoperative sagittal MRI demonstrating a posterior epidural abscess extending across multiple spinal levels with significant spinal cord compression. (B) Preoperative axial MRI confirming circumferential epidural collection and canal compromise. (C) Postoperative sagittal MRI following limited decompression and catheter-directed epidural irrigation showing resolution of the epidural collection. (D) Postoperative axial MRI demonstrating adequate decompression of the spinal canal. SEA, spinal epidural abscess

MRI of the spine demonstrated an extensive posterior epidural abscess extending from C2 to L3, producing significant spinal cord and cauda equina compression (Figure 1). The imaging findings were consistent with a multilevel spinal epidural collection causing substantial canal compromise.

Given the extent of disease and the presence of neurological symptoms, urgent surgical decompression was undertaken. A single-level L1-L2 laminectomy was performed. Upon opening the epidural space, purulent material was encountered under pressure, confirming the diagnosis of epidural abscess.

To facilitate drainage across the extensive cranio-caudal extent of the infection, an 8-French pediatric feeding tube was introduced into the epidural space and carefully advanced both cranially and caudally. Copious saline irrigation was performed through the catheter until clear effluent was obtained, allowing evacuation of purulent material from multiple spinal levels while avoiding extensive multilevel laminectomy.

Intraoperative cultures subsequently grew methicillin-sensitive *Staphylococcus aureus* (MSSA). The patient was commenced on targeted antimicrobial therapy (flucloxacillin 2 g IV four times daily) for six weeks following consultation with infectious disease specialists.

Postoperatively, the patient demonstrated rapid neurological improvement with progressive recovery of lower-limb function over approximately two weeks. Bladder function improved, and inflammatory markers gradually normalized during the postoperative course. At the six-week follow-up, the patient had achieved complete neurological recovery with no residual deficits.

## Discussion

Methods

**Figure 2:**
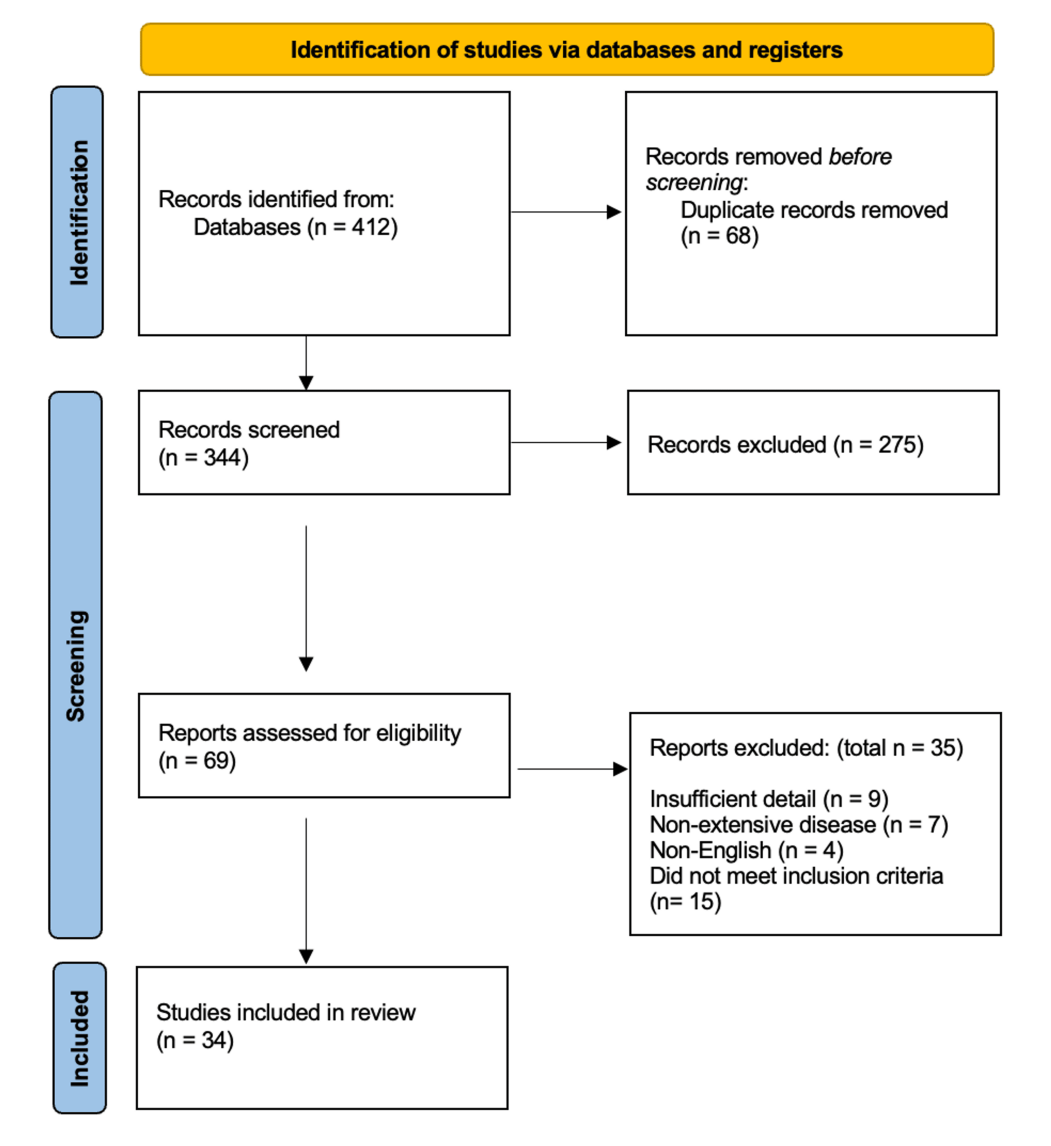
PRISMA 2020 flow diagram of the spinal epidural abscess systematic review (January 1991-March 2025)

This systematic review was conducted in accordance with the PRISMA 2020 statement (Figure 2) [8].

A structured literature search was developed and peer-reviewed in accordance with the PRESS guideline [9]. Searches were performed across MEDLINE (via PubMed), EMBASE, Scopus, the Cochrane Library, and Google Scholar for studies published between January 1991 and March 2025. The complete search string used in PubMed was ("spinal epidural abscess") AND ("extensive" OR "holospinal" OR "panspinal") AND ("laminectomy" OR "laminotomy" OR "skip laminectomy" OR "irrigation" OR "catheter").

Eligible studies included case reports and case series describing surgically managed extensive or holospinal SEA. Extensive disease was defined as continuous or near-continuous involvement of three or more spinal regions, consistent with prior definitions in the literature [4]. Studies were required to report operative details and postoperative neurological outcomes. Exclusion criteria included localized single-level abscess, exclusively nonoperative management, review articles without original patient data, and non-English publications without extractable information. Additionally, studies for which the full text could not be retrieved were excluded, which may introduce selection bias.

Titles and abstracts were independently screened by two reviewers, followed by full-text assessment of potentially eligible studies. Disagreements were resolved by consensus between the two reviewers; a third reviewer was available for arbitration if consensus could not be reached, though this was not required. No formal quality assessment or risk-of-bias tool was applied, as validated instruments for case reports and case series are limited; this represents an acknowledged limitation. Extracted data included patient demographics, comorbidities, microbiological findings, radiological extent of disease, surgical approach, use and type of epidural irrigation, antimicrobial therapy, neurological outcomes, and duration of follow-up. Due to heterogeneity in study design, outcome reporting, and follow-up duration, quantitative meta-analysis was not feasible.

An institutional case was included as an illustrative example to demonstrate the contemporary application of single-level decompression with catheter-directed epidural irrigation, but was excluded from the systematic analysis.

Study Selection

The systematic search identified 412 records across all databases. After the removal of 68 duplicates, 344 titles and abstracts were screened. Of these, 275 were excluded for irrelevance to extensive SEA or absence of surgical management. Sixty-nine full-text articles were assessed for eligibility, of which 35 were excluded: nine for insufficient clinical detail, seven for localized (non-extensive) disease, four as non-English publications with no extractable data, and 15 for failure to meet additional inclusion criteria (operative details or postoperative neurological outcomes not reported). A total of 34 studies were included in the qualitative synthesis (Figure 2).

Patient Demographics and Comorbidities

Across the 34 included studies, patients were predominantly male (approximately 70% of reported cases), consistent with prior epidemiological reports of SEA [1,10]. Age at presentation ranged from infancy to the eighth decade of life, although most patients were middle-aged or older adults (median age approximately 50 years, where it was reported). Diabetes mellitus was the most frequently reported comorbidity, identified in approximately 40% of cases, followed by immunosuppression (including corticosteroid use and malignancy), IV drug use, and chronic renal disease. Comorbidity data were not available for all included studies.

Microbiological Findings

Microbiological data were available in the majority of included cases (approximately 80%). *S. aureus *was the most commonly isolated pathogen, accounting for approximately 60% of cases with available data, including both MSSA and methicillin-resistant *Staphylococcus aureus *strains [1,11]. Streptococcal species represented the next most frequent group, while Gram-negative organisms, including *Escherichia coli *and *Klebsiella pneumoniae*, were less commonly identified. Polymicrobial infection was rarely reported (fewer than 5% of cases).

Radiological Extent and Abscess Distribution

All included cases demonstrated extensive disease, defined as involvement of three or more spinal regions or continuous holospinal spread. Abscesses most frequently extend across the cervical and thoracolumbar regions. Dorsal epidural collections were more commonly reported than ventral or circumferential involvement (approximately 65% vs. 35%), although mixed patterns were also described. Concomitant osteodiscitis, paraspinal abscesses, and psoas collections were frequently noted.

Surgical Strategies

Multiple surgical strategies have been described for extensive SEA, including skip laminectomy, laminotomy, minimally invasive tubular approaches, and catheter-directed epidural irrigation [14-40]. Continuous multilevel laminectomy was rarely performed. Instead, most authors reported the use of limited decompression techniques, including skip laminectomies, laminotomies, unilateral laminotomy for bilateral decompression, or minimally invasive tubular or endoscopic approaches [7,12,15-19,24]. These approaches were commonly combined with catheter-directed epidural irrigation to allow clearance of purulent material across multiple spinal segments while preserving posterior elements.

A wide range of irrigation devices was utilized, including epidural catheters, ventricular catheters, ureteric catheters, nasogastric tubes, and pediatric feeding tubes [17,22]. Irrigation volumes ranged from 500 mL to several liters of normal saline per session, where reported, with repeated passes until clear effluent was obtained. Instrumented stabilization was infrequently required and was typically reserved for cases with pre-existing or iatrogenic instability.

Neurological Outcomes

Postoperative neurological outcomes were reported in most studies. Over 80% of patients demonstrated neurological improvement following surgical intervention, ranging from partial recovery to complete resolution of deficits. Several reports documented recovery from severe preoperative neurological impairment, including paraplegia or quadriplegia. Poor neurological outcomes were uncommon and were generally associated with delayed presentation, severe preoperative deficits, or significant medical comorbidity.

Overall, the included studies demonstrate consistent trends in the presentation and management of extensive SEA. Patients were predominantly male with a high prevalence of medical comorbidities, most notably diabetes mellitus. *S. aureus *remained the dominant causative organism across all decades. Surgically, the literature reflects a clear preference for limited or skip decompressive techniques combined with catheter-directed epidural irrigation rather than continuous multilevel laminectomy. Where neurological outcomes were reported, most patients experienced meaningful postoperative improvement, though the retrospective and heterogeneous nature of the included studies warrants caution in generalizing these findings. Table 1 summarizes all included studies.

**Table 1:** Published cases of surgically managed extensive SEA (1994-2025) Outcome categories reflect reporting in the original studies and were not uniformly defined. Extensive SEA was defined as involvement of three or more spinal regions or continuous multilevel disease. IVDU, intravenous drug use; MRSA, methicillin-resistant *Staphylococcus aureus*; MSSA, methicillin-sensitive *Staphylococcus aureus*; SEA, spinal epidural abscess; ULBD, unilateral laminotomy for bilateral decompression

Study	Age/sex	Comorbidities	Abscess extent	Pathogen	Surgical approach	Irrigation	Outcome	Reference
Abd-El-Barr et al. (2015)	51 M	Diabetes	C2-S2	MSSA	Skip laminotomies	Yes	Full recovery	[1]
Ahuja et al. (2019)	18 M	None	C1-S5	MRSA	T2 and T10 laminectomies	Yes	Improved	[2]
AlQahtani et al. (2025)	59 F	Not reported	Extensive	Not reported	Single limited incision	Yes	Improved	[3]
Burton et al. (2014)	30 F	IVDU, pregnancy	C1-S1	MSSA	T11-L1 laminectomies	Yes	Full recovery	[5]
Dang and Rajkumar (2018)	55 F	Diabetes	C1-L4	Klebsiella	Multilevel decompression	Yes	Improved	[6]
Denis et al. (2019)	53 F	Septic arthritis	L1-S1	MSSA	L5 laminectomy	Yes	Full recovery	[10]
Hugues Dokponou et al. (2024)	54 M	None	C7-T10	MSSA	Targeted laminectomy	Yes	Improved	[11]
Elsamaloty et al. (2010)	53 M	Diabetes	C1-L1	MSSA	Multilevel laminectomies	Yes	Improved	[12]
Fujii et al. (2020)	81 M	Not stated	Extensive	Not stated	Percutaneous drainage	Yes	Improved	[13]
Gorchynski et al. (2009)	33 M	IVDU	C1-L5	MRSA	Segmental laminectomies	Yes	Improved	[14]
Hagel et al. (2023)	67 M	None	T9-L4	Streptococcus pneumoniae	Endoscopic + open	Yes	Full recovery	[15]
Halalmeh et al. (2022)	48 M	Diabetes	C1-S1	MSSA	Skip laminectomies	Yes	Improved	[16]
Kim et al. (2021)	79 F	Diabetes, RA	T3-L5	MRSA	Laminotomies	Yes	Full recovery	[17]
Lau et al. (2014)	50 M	Diabetes	C1-S1	MSSA	Transoral + posterior	Yes	Improved	[18]
Lee et al. (2022)	58 M	Diabetes	C1-L5	Escherichia coli	ULBD	Yes	Full recovery	[19]
Leonard and Kaufman (2001)	Neonate	Teratoma	C1-L5	Polymicrobial	Laminotomy	Yes	Full recovery	[20]
Lin et al. (2013)	41 M	Diabetes	C1-S1	Staphylococcus capitis	None	No	Lost follow-up	[21]
O'Brien et al. (2011)	71 M	Diabetes	C1-S1	MSSA	Conservative	No	Improved	[22]
Oh et al. (2016)	19 M	Pyomyositis	C2-S1	MSSA	Laminotomies	Yes	Full recovery	[23]
Parkinson and Sekhon (2004)	48 F	None	C1-S1	Streptococcus	Laminectomy	No	Partial recovery	[24]
Pi et al. (2023)	70 M	Diabetes	C2-L4	Staphylococcus epidermidis	Hemilaminectomy	Yes	Improved	[25]
Rajpal et al. (2021)	70 F	Pneumonia	C2-S3	MRSA	Selective laminectomy	Yes	Full recovery	[26]
Roberti (2020)	Not stated, M	Not stated	Holocord	Not stated	Tubular decompression	Yes	Improved	[27]
Saito et al. (2019)	60 F	Not stated	Extensive	Not stated	Multilevel drainage	Yes	Improved	[28]
Shoakazemi et al. (2013)	44 M	Psoas abscess	C1-S1	MSSA	Multilevel laminectomy	Yes	Improved	[30]
Simpson et al. (1991)	63 M	RA	C2-S2	Unknown	None	No	Death	[31]
Sinatra and Alander (2017)	15 M	Lemierre syndrome	T1-L4	Fusobacterium	Hemilaminectomy	Yes	Full recovery	[32]
Smith and Kavar (2010)	25 M	Crohn disease	C2-S1	Not reported	Laminotomies	Yes	Partial recovery	[33]
Supreeth and Al Ghafri (2019)	59 F	Diabetes	C1-L5	S. pneumoniae	Hemilaminectomy	Yes	Full recovery	[34]
Tahir et al. (2010)	38 F	IVDU	C1-S1	MSSA	Laminotomies	Yes	Full recovery	[35]
Thomson (2018)	66 F	Atrial flutter	C1-L4	MSSA	Skip laminotomy	Yes	Full recovery	[37]
Tracz et al. (2022)	74 M	Diabetes	C2-S1	Cutibacterium acnes	Laminectomy	Yes	Not stated	[38]
Van Bergen et al. (2009)	50 M	None	C2-L3	MSSA	Conservative	No	Full recovery	[39]
Xiang et al. (2016)	65 F	Diabetes	C1-S2	MRSA	Laminotomies	Yes	Improved	[40]

This systematic review highlights evolving surgical strategies for the management of extensive SEA. The predominance of limited decompression combined with catheter-directed epidural irrigation reflects a shift away from traditional extensive laminectomy, driven by concerns regarding surgical morbidity, spinal instability, and prolonged recovery [7,10,13]. Favorable neurological outcomes reported across heterogeneous case series support the effectiveness of this approach in achieving adequate source control while preserving spinal integrity, though these findings must be interpreted with caution given the retrospective nature of the data [15,18,19].

While traditional multilevel laminectomy remains effective for focal disease, its application in extensive SEA is limited by the risks of postoperative instability and blood loss [7,10,13]. Alternative strategies, including skip laminectomies, laminotomies, unilateral laminotomy for bilateral decompression, and minimally invasive tubular or endoscopic techniques, have therefore been described [15,18,19,24,27].

Endoscopic and minimally invasive approaches have shown favorable outcomes in selected patients, particularly those with significant medical comorbidities or ventrally located collections [13,27]. However, these techniques require specialized expertise and may not be universally available. In contrast, limited open decompression combined with catheter-directed epidural irrigation represents a widely applicable and reproducible strategy adaptable to varying anatomical and pathological scenarios [17,19,22].

Limitations

This review is limited by reliance on retrospective case reports and small case series, introducing heterogeneity and potential publication bias. Studies restricted to English publications with extractable data may further bias results toward centers with greater reporting activity. Standardized neurological outcome measures were inconsistently reported, precluding quantitative synthesis [6,17]. No formal risk-of-bias assessment was applied, as validated tools for case-level evidence are limited. Nevertheless, aggregation of these rare cases provides valuable guidance for clinicians managing extensive SEA.

## Conclusions

Extensive SEA remains a rare but potentially devastating condition that requires prompt recognition and timely intervention. This systematic review demonstrates that contemporary management has shifted away from traditional extensive multilevel laminectomy toward targeted decompressive strategies combined with catheter-directed epidural irrigation and prolonged antimicrobial therapy. Across heterogeneous case reports and series, these approaches were associated with favorable neurological outcomes in over 80% of patients while minimizing surgical morbidity and the risk of postoperative spinal instability, though the evidence base is limited to retrospective case-level data.

Given the rarity of extensive disease and the absence of high-level comparative studies, operative management should be individualized, taking into account neurological status, abscess extent, anatomical considerations, and patient comorbidities. Further multicenter collaboration and standardized outcome reporting are required to refine treatment algorithms and optimize outcomes for patients with this challenging condition.
